# Selection and characterization of bacteriophages specific to *Salmonella* Choleraesuis in swine

**DOI:** 10.14202/vetworld.2022.2856-2869

**Published:** 2022-12-16

**Authors:** Pattaraporn Sriprasong, Napakhwan Imklin, Rujikan Nasanit

**Affiliations:** Department of Biotechnology, Faculty of Engineering and Industrial Technology, Silpakorn University, Nakhon Pathom 73000, Thailand

**Keywords:** antimicrobials, bacteriophage, biocontrol, *Salmonella* Choleraesuis, swine

## Abstract

**Background and Aim:**

*Salmonella* Choleraesuis is the most common serotype that causes salmonellosis in swine. Recently, the use of bacteriophages as a potential biocontrol strategy has increased. Therefore, this study aimed to isolate and characterize bacteriophages specific to *S*. Choleraesuis associated with swine infection and to evaluate the efficacy of individual phages and a phage cocktail against *S*. Choleraesuis strains in simulated intestinal fluid (SIF).

**Materials and Methods:**

Three strains of *S*. Choleraesuis isolated from pig intestines served as host strains for phage isolation. The other 10 *Salmonella* serovars were also used for the phage host range test. The antibiotic susceptibility of the bacterial strains was investigated. Water samples from natural sources and drain liquid from slaughterhouses were collected for phage isolation. The isolated phages were characterized by determining the efficiency of plating against all Salmonella strains and the stability at a temperature range (4°C–65°C) and at low pH (2.5–4.0) in simulated gastric fluids (SGFs). Furthermore, morphology and genomic restriction analyses were performed for phage classification phages. Finally, *S*. Choleraesuis reduction in the SIF by the selected individual phages and a phage cocktail was investigated.

**Results:**

The antibiotic susceptibility results revealed that most *Salmonella* strains were sensitive to all tested drugs. *Salmonella* Choleraesuis KPS615 was multidrug-resistant, showing resistance to three antibiotics. Nine phages were isolated. Most of them could infect four *Salmonella* strains. Phages vB_SCh-RP5i3B and vB_SCh-RP61i4 showed high efficiency in infecting *S*. Choleraesuis and *Salmonella* Rissen. The phages were stable for 1 h at 4°C–45°C. However, their viability decreased when the temperature increased to 65°C. In addition, most phages remained viable at a low pH (pH 2.5–4.0) for 2 h in SGF. The efficiency of phage treatment against *S*. Choleraesuis in SIF showed that individual phages and a phage cocktail with three phages effectively reduced *S*. Choleraesuis in SIF. However, the phage cocktails were more effective than the individual phages.

**Conclusion:**

These results suggest that the newly isolated phages could be promising biocontrol agents against S. Choleraesuis infection in pigs and could be orally administered. However, further *in vivo* studies should be conducted.

## Introduction

The incidence of foodborne diseases remain high globally, directly impacting human health. Globally, 600 million people get sick from foodborne diseases, and 420,000 people die yearly [1]. *Salmonella* spp. is the most common cause of bacterial foodborne outbreaks. Recently, the European Food Safety Authority and the European Centre for Disease Prevention and Control reported 694 foodborne outbreaks of *Salmonella*, with 3686 cases of illnesses, 812 hospitalizations, and seven mortality in 22 European Union member states in 2020 [2]. As *Salmonella* generally colonizes the gastrointestinal tract and is excreted in feces, this can cause cross-contamination in raw foods of animal origin during production and slaughter [3]. *Salmonella* has been the most widely reported swine pathogen in global trends in infectious diseases of swine and has significantly impacted the productivity of the swine industry, globally [4]. *Salmonella* can infect swine during transport to slaughterhouses or at lairage depending on variable factors, such as stress, environmental contamination, and dose-response parameters [5]. The porcine *Salmonella* can be divided into two groups based on its host range and clinical presentation. The first group consisted of *Salmonella* *enterica* serovar Choleraesuis, which is a host-adapted serovar and causes systemic diseases. The second group included other *Salmonella* serovars, such as *Salmonella* Typhimurium. The latter group has a broader host range and is likely to elicit transient enteritis [6]. Moreover, *Salmonella* contamination has frequently been reported in animal feeds, raw pork, pork products, pig carcasses, and slaughterhouses [7–10]. *Salmonella* Choleraesuis is the most frequent serotype found in swine [11]. In the United States, *S*. Choleraesuis infections have been reported in more than 90% of swine salmonellosis cases. Furthermore, swine infected with *S*. Choleraesuis can lead to a contaminated environment, food, or water sources, which can be a reservoir for *S*. Choleraesuis infection in humans [12]. *Salmonella* Choleraesuis can cause severe systemic illnesses and extraintestinal infections with high mortality rates in humans [13, 14]. Serotype Choleraesuis usually causes septicemia in swine, characterizable by hepatitis, pneumonia, and cerebral vasculitis [15]. In general, it can cause disease in both young and older swine [16]. However, it is more common in younger swine than in older swine [17].

Antibiotics are widely used in the swine industry to prevent and treat infectious diseases. However, antibiotic abuse can result in antibiotic-resistant bacteria. These bacteria can survive and widely spread environmentally resistant genes [18]. In addition, numerous reports of antibiotic-resistant *Salmonella* have been discovered on swine farms [19–23]. The antibiotic-resistant *Salmonella* associated with swine affects swine production as well as human health through direct contact with animals, the food chain, and the environment [24]. These human health consequences might cause significant therapeutic challenges associated with a longer duration of illness and higher mortality rates [25]. Therefore, developing a potent antibacterial alternative to control these bacterial infections is crucial.

Bacteriophages or phages are one of the promising alternatives for reducing *Salmonella* prevalence from farm to fork. Phage therapy positively affects both animal and human health. Phages can also decrease the distribution of antibiotic resistance bacteria in various environments and treat bacterial infections, including multidrug-resistant bacteria [26]. However, the efficiency of therapy differs according to the complexity of the bacterial target and infection site [27]. Each phage differs in the host range. Therefore, selecting a broad host range phage is necessary and useful for phage applications that can infect multiple species of bacteria [28]. Conversely, phage cocktails have been used to treat infections caused by various bacterial strains. This approach could be useful in tackling bacteria with resistance to a certain phage [29, 30]. Several studies have shown that phages can control *Salmonella* infection in swine [31–34].

Therefore, this study aimed to isolate and characterize phages specific to *S*. Choleraesuis isolated from the infected swine intestines and to evaluate the efficiency of individual phage and phage cocktail treatment against *S*. Choleraesuis in simulated intestinal fluid (SIF) as a guideline for further phage treatment in swine intestine conditions.

## Materials and Methods

### Ethical approval

This study required no ethical approval because no animals were used.

### Study period and location

This study was conducted from July 2019 to August 2022 at the Department of Biotechnology, Faculty of Engineering and Industrial Technology, Silpakorn University, Sanam Chandra Palace Campus, Nakhon Pathom, Thailand.

### Bacterial strains and culture conditions

The *Salmonella* strains used in this study are listed in Table-1 along with the source. Three strains of *Salmonella* Choleraesuis, including KPS585, KPS604-1, and KPS615, obtained from the Veterinary Diagnostic Laboratory, Faculty of Veterinary Medicine, Kasetsart University Kamphaeng Saen Campus, were isolated from *Salmonella*-infected swine intestines. These bacterial strains served as hosts for phage isolation in this study. Bacterial strains were cultured at 37°C overnight in Tryptic Soy Broth (TSB) before the experiment and maintained at −80°C in 20% glycerol.

**Table-1:** Antibiotic susceptibility of *Salmonella* strains.

*Salmonella* strain	Antimicrobial agent					

	GEN (10 µg)	ENR (5 µg)	CST (10 µg)	SXT (25 µg)	NEO (30 µg)	KAN (30 µg)
*S*. Choleraesuis KPS585	R	S	S	S	S	S
*S*. Choleraesuis KPS604-1	S	S	S	S	I	S
*S*. Choleraesuis KPS615	S	R	S	S	R	R
*S*. Anatum DMST50705	S	S	S	S	S	S
*S*. Corvallis DMST34495	S	S	S	S	I	S
*S*. Enteritidis DMST8536	S	S	S	S	S	S
*S*. Hadar DMST10634	S	S	S	S	S	S
*S*. Lexington DMST50707	S	S	S	S	S	S
*S*. Rissen DMST7097	S	S	S	S	S	S
*S*. Stanley DMST16874	S	S	S	S	S	S
*S*. Typhimurium ATCC13311 DMST562	S	S	S	S	S	S
*S*. Weltevreden DMST15677	S	S	S	S	S	S
*S*. Worthington DMST50712	S	S	S	R	I	S

KPS=Kasetsart University, Kamphaeng Saen Campus, DMST=Department of Medical Sciences, Thailand, GEN=Gentamicin, ENR, Enrofloxacin, CST=Colistin Sulfate, SXT=Sulfamethoxazole, NEO=Neomycin, KAN=Kanamycin, S is susceptible, I is intermediate resistant, and R is resistant. *S*. Choleraesuis=*Salmonella* Choleraesuis, *S*. Anatum=*Salmonella* Anatum, *S*. Corvallis=*Salmonella* Corvallis, *S*. Enteritidis=*Salmonella* Enteritidis, *S*. Hadar=*Salmonella* Hadar, *S*. Lexington=*Salmonella* Lexington, *S*. Rissen=*Salmonella* Rissen, *S*. Stanley=*Salmonella* Stanley, *S*. Typhimurium=*Salmonella* Typhimurium, *S*. Weltevreden=*Salmonella* Weltevreden, *S*. Worthington=*Salmonella* Worthington

### Antibiotic susceptibilities to *Salmonella* strains

The antibiotic susceptibilities of *Salmonella* strains were determined using the disk diffusion method according to the Clinical and Laboratory Standards Institute (CLSI) guidelines [35]. Six antimicrobial disks, including enrofloxacin (ENR) (5 μg), neomycin (NEO) (30 μg), colistin sulfate (CST) (10 μg), sulfamethoxazole (SXT) (25 μg), kanamycin (KAN) (30 μg), and gentamicin (GEN) (10 μg), were tested. The bacterial culture (equivalent to 0.5 McFarland) was spread onto a Tryptic Soy Agar (TSA) plate. Then, the culture was left to dry for 3–5 min. Antibiotic disks were placed on the TSA surface. After incubation at 37°C for 16–18 h, the zone of inhibition was observed and interpreted according to the CLSI breakpoint.

### Phage isolation, purification, and propagation

Four water samples were collected from the irrigation canals, Sa Bua in Nakhon Pathom and Sa Kaeo at Silpakorn University, Sanam Chandra Palace Campus, and drain liquid samples were collected from slaughterhouses for phage isolation. *Salmonella* Choleraesuis KPS585, KPS604-1, and KPS615 served as the host strains. Briefly, the samples were centrifuged at 3000× *g* for 10 min to remove large particulates. The supernatant was mixed with each culture strain and 10X concentrated TSB medium in a ratio of 9:0.1:1. The mixture was incubated at 37°C overnight. Then, the enriched culture was centrifuged at 12,500× *g* for 10 min and filtered using a polyethersulfone syringe filter with a 0.22 μm pore size. The spot test was performed to primarily screen for the presence of certain phages in the samples. A bacterial lawn was prepared by adding 100 μL overnight bacterial host and mixed with 3.5 mL molten agar (TSA with 0.45% w/v agar) and overlaid immediately onto the TSA plate. Ten microliters of each lysate were spotted on the bacterial lawn and incubated overnight at 37°C. The samples that produced the lysis zone were selected for phage isolation using an agar overlay assay. For further phage purification, the individual plaques with different morphologies both in size and appearance were collected and suspended in an SM buffer.

The isolated phages were purified using an agar overlay assay by taking 100 μL of phage samples resuspended in the SM buffer mixed with 100 μL of the host culture and added to 3.5 mL molten agar. The mixture was poured onto the TSA plate. The plates were allowed to dry at room temperature (25°C) for 10 min and incubated overnight at 37°C. Plaques with different morphologies obtained from each host were collected and resuspended in 1 mL of the SM buffer. The tubes were left at 25°C for at least 30 min, allowing the phage particles to diffuse into the solution. The purification process was repeated three times ensuring successful phage purification. The purified phages were propagated with their hosts to prepare high-titer stocks. One hundred microliters of the purified phage suspension were mixed with 100 μL of an overnight host culture in 3.5 mL molten agar and poured onto the TSA plate. The plates were incubated at 37°C overnight. The top agar containing a high density of plaques was scraped off using a sterile spreader and transferred into a centrifuge tube. The remaining phages in the agar plate were collected by adding 2 mL of the TSB and pipetted into the same tube. The tubes were maintained at 25°C for at least 30 min. The suspension was centrifuged at 6000× *g* for 20 min at 4°C and filtered. The phage titer was determined as a plaque-forming unit per milliliter (PFU/mL) using agar overlay assay and stored at 4°C for further use.

### Efficiency of plating (EOP)

Agar overlay assay was used to evaluate all phages, analyzing the effectiveness of each phage against a range of the target bacteria (Table-1). The EOP value was calculated using the average phage titer obtained from the target bacterium divided by the average phage titer obtained from its host. The efficiency of phages against the target bacteria was classified as high (EOP > 0.5), moderate (EOP > 0.2–0.5), low (EOP > 0.001–0.2), and inefficient (EOP < 0.001) with regard to the EOP values [36]. This assay was performed in triplicates.

### Temperature stability

Temperature stability tests were performed by incubation at 4°C, 28°C, 37°C, 45°C, and 65°C. One hundred microliters of each phage were added to 900 μL TSB medium pre-incubated at the particular temperatures. The mixture was incubated at those specific temperatures for 1 h and immediately diluted in the SM buffer before phage titer determination. Each experiment was done in triplicate.

### Low pH stability

Phages can become inactive and be destroyed due to exposure to low pH in swine gastric juices. Therefore, in this study, the phages were evaluated using the simulated gastric fluid (SGF) by simulating different pH encountered along the gastric of swine. The SGF consisted of 34 mM NaCl and 3.2 mg/mL pepsin at pH 2.5, 3.0, 3.5, and 4.0 [37]. One hundred microliters of each phage (10^9^ PFU/mL) were added to 9.9 mL pre-warmed (37°C) SGF and incubated at 37°C in a shaking incubator for 1 and 2 h. After incubation, the phage titer was determined. The experiments were performed in triplicate.

### Transmission electron microscopy (TEM)

The purified phage lysate (≥10^9^ PFU/mL) was dropped on a formvar-coated copper grid. Negative staining was conducted using 2% uranyl acetate. The electron micrographs were taken under a Hitachi High-Tech HT7700 transmission electron microscope (Japan) at a voltage of 80 kV, at the Scientific Equipment and Research Division, KURDI, Kasetsart University.

### Restriction analysis of phage DNA

One milliliter of purified phage suspension (>10^9^ PFU/mL) was treated with 1 μL nuclease enzymes (1 mg/mL DNase and 10 mg/mL RNase final concentration) to degrade bacterial nucleic acids. Then, 12.5 μL of 1 M MgCl_2_ was added and inversely mixed. The mixture was incubated at 37°C for 30 min. After incubation, 40 μL of 0.5 M EDTA, 10 μL 20 mg/mL proteinase K, and 50 μL 10% SDS were added to the mixture, then incubated at 55°C for 1.5 h. The sample was mixed with an equal volume of phenol/chloroform/isoamyl alcohol (25:24:1). After centrifugation at 11,300× *g* for 10 min, the aqueous phase was transferred to a new microfuge tube. Then, 0.1 volume of 3 M sodium acetate buffer (pH 5.2) and 2.5 volumes of cold ethanol were added, mixed thoroughly, and incubated at −20°C for 2 h. Next, the mixture was centrifuged and the supernatant was decanted. The nucleic acid was precipitated with 1 mL 70% ethanol. After centrifugation, the supernatant was decanted, and the pellet was dried at 25°C. The nucleic acid was dissolved in sterilized deionized water.

Phage DNA samples were digested using restriction enzymes, *Eco*RI, *Eco*RV, and *Hin*fI, following the manufacturer’s recommendation. The DNA fragments were separated using 1% agarose gel electrophoresis in 1× TAE buffer.

### The efficiency of phage treatment against *S*. Choleraesuis in SIF

The SIF was prepared by adding 10 mg/mL pancreatin and 20 mg/mL bile salt to 50 mM KH_2_PO_4_ at pH 6.8 [38]. The individual phages; vB_SCh-RP5i3B, 60i4A, and 61i4, and a cocktail of three phages were diluted in an SM buffer to obtain a final concentration of 10^7^ PFU/mL and 10^8^ PFU/mL. The culture of each bacterial strain (*S*. Choleraesuis KPS585, KPS604-1, and KPS615) at OD_600_ of 0.1 (approximately 10^7^ CFU/mL) was mixed with each phage or a phage cocktail to obtain a multiplicity of infection (MOI) of 1 and 10 in a volume of 30 mL. The mixture was incubated at 37°C with shaking at 125 rpm. The samples were collected at 10 min intervals for 120 min. The SM buffer was used instead of the phage lysate for the control experiments. The diluted samples were spotted on TSA plates to enumerate the viable counts of *Salmonella*.

### Statistical analysis

Statistical analysis was performed using SPSS statistics version 23.0 (IBM Corp., Armonk, NY, USA). The stability of phages at various temperatures between the initial titer and the titer after 1 h of exposure was compared using the Student’s t-test. In addition, one-way analysis of variance (ANOVA) was conducted to evaluate the difference in phage stability at different temperatures, phage stability after exposure at low pH for 1 and 2 h, and bacterial reduction by phage treatment at different MOIs at each time point. Two-way ANOVA was used to estimate the effect of pH, time, and the interaction between pH and time on phage stability. Turkey’s Honestly Significant Different test was used to compare the means pair-wise. Differences at the level of p < 0.05 were consid-ered statistically significant.

## Results

### Antibiotic susceptibility to *Salmonella* strains

The results of antibiotic susceptibility of all *Salmonella* strains are shown in Table-1. These strains were sensitive to CST. Three *Salmonella* strains were resistant to some antibiotics. These included *S*. Choleraesuis KPS585, which was GEN resistant, *S*. Choleraesuis KPS615, which was ENR, NEO, and KAN resistant, and *Salmonella* Worthington, which was SXT resistant. Furthermore, *S*. Choleraesuis KPS604-1, *Salmonella* Corvallis, and *S*. Worthington showed intermediate resistance to NEO (Table-1).

### Bacteriophages isolated from natural and drainage from the slaughterhouse

Nine phages were isolated from the samples obtained from the irrigation canals and slaughterhouses, with *S*. Choleraesuis strains serving as hosts. Among these phages, six were isolated from an irrigation canal and three were isolated from slaughterhouses using *S*. Choleraesuis KPS585, KPS604-1, and KPS615 as host strains (Table-2). The isolated phages differed in plaque size and produced halos around their plaques. Among these phages, phage vB_SCh-RP5i3B formed the smallest and most clear plaques surrounded by translucent halos with a diameter of <1 mm, while vB_SCh-RP5i3A, vB_SCh-RP60i3A, vB_SCh-RP60i3B, vB_SCh-RP60i3C, vB_SCh-RP60i4A, vB_SCh-RP60i4B, vB_SCh-RP61i3, and vB_SCh-RP61i4 formed clear plaques surrounded by translucent halos with a diameter of 1.0 mm–4.0 mm (Table-2).

**Table-2:** Morphological characteristics of bacteriophages.

Bacteriophages	Host strain	Plaque morphology	Phage morphology (nm)*		

			Head width	Tail width	Tail length
vB_SCh-RP5i3A	*S*. Choleraesuis KPS585	Clear with halo; ø 4 mm.	55.00 ± 4.92	9.60 ± 2.27	8.00 ± 2.67
vB_SCh-RP5i3B	*S*. Choleraesuis KPS585	Clear with halo; ø <1 mm.	48.85 ± 1.86	7.69 ± 0.00	99.23 ± 3.03
vB_SCh-RP60i3A	*S*. Choleraesuis KPS604-1	Clear with halo; ø 4 mm.	52.50 ± 3.23	12.50 ± 0.00	11.25 ± 2.64
vB_SCh-RP60i3B	*S*. Choleraesuis KPS604-1	Clear with halo; ø 1.5 mm.	51.54 ± 3.72	7.69 ± 0.00	113.08 ± 5.19
vB_SCh-RP60i3C	*S*. Choleraesuis KPS604-1	Clear with halo; ø 1 mm.	47.69 ± 3.24	7.69 ± 0.00	93.08 ± 5.68
vB_SCh-RP60i4A	*S*. Choleraesuis KPS604-1	Clear with halo; ø 3 mm.	48.46 ± 1.99	7.69 ± 0.00	91.15 ± 2.60
vB_SCh-RP60i4B	*S*. Choleraesuis KPS604-1	Clear with halo; ø 1.5 mm.	51.54 ± 1.99	6.54 ± 1.86	3.85 ± 0.00
vB_SCh-RP61i3	*S*. Choleraesuis KPS615	Clear with halo; ø 3 mm.	66.54 ± 1.86	11.54 ± 0.00	126.15 ± 3.03
vB_SCh-RP61i4	*S*. Choleraesuis KPS615	Clear with halo; ø 2 mm.	53.46 ± 1.22	7.69 ± 0.00	124.23 ± 3.65

* The average sizes of head and tail phages were calculated by measuring at least 10 particles of each phage. *S*. Choleraesuis=*Salmonella* Choleraesuis

### The EOP of the isolated phages

The EOP results revealed that all phages could affect other *Salmonella* serovars other than their host (Table-3). Some *Salmonella* serovars were efficiently infected with vB_SCh-RP5i3B and vB_SCh-RP61i4. vB_SCh-RP5i3B had a high efficiency (EOP > 0.5) in infecting all strains of *S*. Choleraesuis and *Salmonella* Rissen. However, it was inefficient (EOP < 0.001) for *Salmonella* Hadar. In addition, vB_SCh-RP61i4 could effectively lyse (EOP > 0.5) *S*. Choleraesuis KPS585, *S*. Choleraesuis KPS604-1, and *S*. Rissen. Meanwhile, phages vB_SCh-RP60i3B, vB_SCh-RP60i4A, and vB_SCh-RP61i3 could lyse four tested strains, which were phages vB_SCh-RP60i4A and vB_SCh-RP61i3, exhibited the highest EOP in *S*. Choleraesuis KPS615 and KPS604-1, respectively. Furthermore, phages vB_SCh-RP5i3A, vB_SCh-RP60i3A, vB_SCh-RP60i3C, and vB_SCh-RP60i4B could lyse three tested strains.

**Table-3:** EOP values of bacteriophages.

Phages name	EOP level												

	*S*. Choleraesuis KPS585	*S*. Choleraesuis KPS604-1	*S*. Choleraesuis KPS615	*S*. Anatum	*S*. Corvallis	*S*. Enteritidis	*S*. Hadar	*S*. Lexington	*S*. Rissen	*S*. Stanley	*S*. Typhimurium	*S*. Weltevreden	*S*. Worthington
vB_SCh- RP5i3A	Host	0.427	0.501	-	-	-	-	-	-	-	-	-	-
vB_SCh- RP5i3B	Host	5.682	2.338	-	-	-	10^-5^	-	0.528	-	-	-	-
vB_SCh- RP60i3A	0.941	Host	0.066	-	-	-	-	-	-	-	-	-	-
vB_SCh- RP60i3B	0.003	Host	0.155	-	-	-	-	-	0.034	-	-	-	-
vB_SCh- RP60i3C	0.026	Host	0.827	-	-	-	-	-	-	-	-	-	-
vB_SCh- RP60i4A	0.023	Host	2.240	-	-	-	-	-	0.029	-	-	-	-
vB_SCh- RP60i4B	-	Host	1.189	-	-	-	-	-	0.470	-	-	-	-
vB_SCh- RP61i3	0.051	6.860	Host	-	-	-	-	-	0.059	-	-	-	-
vB_SCh- RP61i4	0.739	1.113	Host	-	-	-	-	-	1.959	-	-	-	-

Phage efficient activity: EOP > 0.5 (high efficiency), EOP > 0.2–0.5 (moderate efficiency), EOP>0.001–0.2 (low efficiency), < 0.001 (inefficiency), -=Not specific. EOP=Efficiency of plating, *S*. Choleraesuis=*Salmonella* Choleraesuis, *S*. Anatum=*Salmonella* Anatum, *S*. Corvallis=*Salmonella* Corvallis, *S*. Enteritidis=*Salmonella* Enteritidis, *S*. Hadar=*Salmonella* Hadar, *S*. Lexington=*Salmonella* Lexington, *S*. Rissen=*Salmonella* Rissen, *S*. Stanley=*Salmonella* Stanley, *S*. Typhimurium=*Salmonella* Typhimurium, *S*. Weltevreden=*Salmonella* Weltevreden, *S*. Worthington=*Salmonella* Worthington

### Stability of bacteriophages at different temperatures

**Figure-1:**
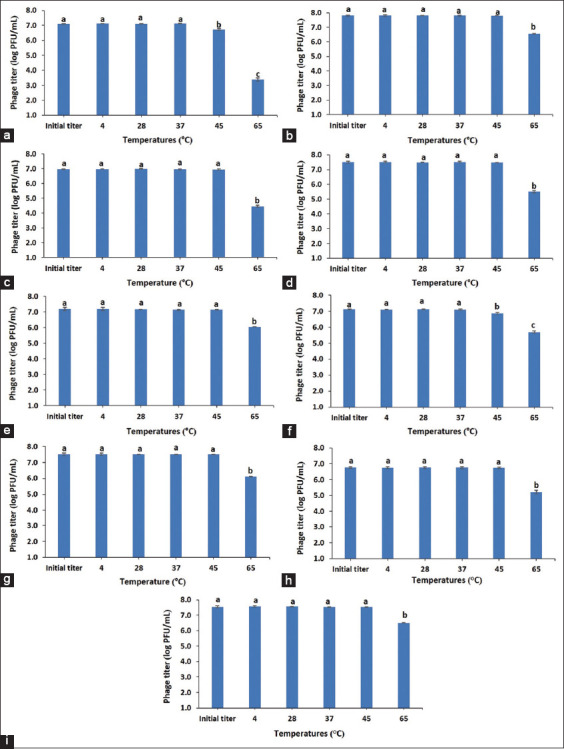
Thermal stability of phages vB_SCh-RP5i3A (a), vB_SCh-RP5i3B (b), vB_SCh-RP60i3A (c), vB_SCh-RP60i3B (d), vB_SCh-RP60i3C (e), vB_SCh-RP60i4A (f), vB_SCh-RP60i4B (g), vB_SCh-RP61i3 (h), and vB_SCh-RP61i4 (i). Data were expressed as mean ± standard deviation for three independent experiments. Data were analyzed using one-way analysis of variance. Different letters above the columns indicate significant differences (p < 0.05).

As shown in Figure-1, all phages were unaffected during storage at 4°C, 28°C, 37°C, and 45°C for 1 h. However, at 45°C, phage vB_SCh-RP5i3A reduced significantly with approximately 0.37 ± 0.08 log PFU/mL (*t*_5_ = 11.776, p < 0.001) compared with the initial titer, while phage vB_SCh-RP60i4A reduced significantly by approximately 0.25 ± 0.04 log PFU/mL (*t*_5_ = 13.922, p < 0.001) compared with the initial titer. The viability of all phages decreased significantly at 65°C with approximately 1.03–3.73 log PFU/mL (p < 0.001).

### Low pH stability

**Figure-2:**
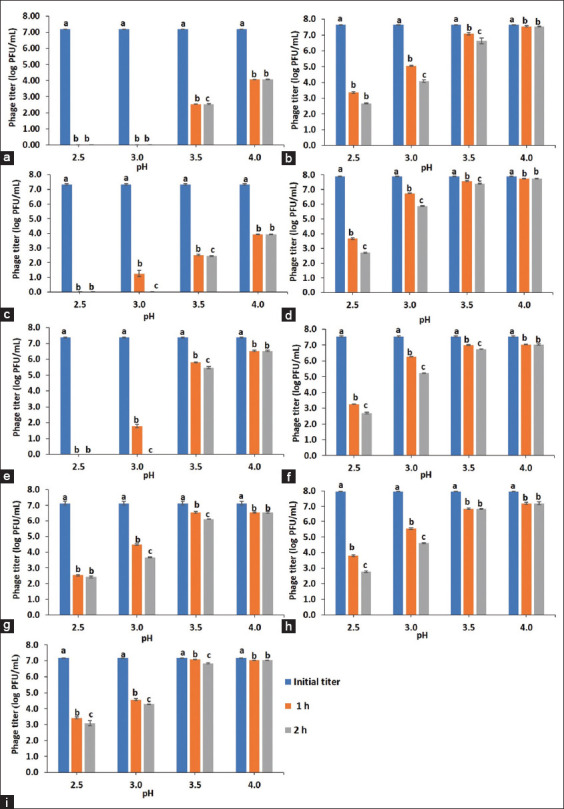
Stability of phages at low pH conditions for 1 and 2 h. (a) Phage vB_SCh-RP5i3A, (b) vB_SCh-RP5i3B, (c) vB_SCh-RP60i3A, (d) vB_SCh-RP60i3B, (e) vB_SCh-RP60i3C, (f) vB_SCh-RP60i4A, (g) vB_SCh-RP60i4B, (h) vB_SCh-RP61i3, and (i) vB_SCh-RP61i4. The results were expressed as mean ± standard deviation for three independent experiments. Different letters above the columns indicate the statistical significance of the difference among the initial and post-incubated phage titer at each pH for 1 and 2 h. Data were performed using one-way analysis of variance followed by Turkey’s Honestly Significant Different at a significance level of p < 0.05.

The phage stability in the SGF at low pH conditions for 1 and 2 h is shown in Figure-2. The viable counts of phage vB_SCh-RP5i3A were significantly influenced by the pH (*F*_3,60_ = 101685.15, p < 0.001), the time (*F*_2,60_ = 752870.03, p < 0.001), and the interaction between pH and time (*F*_6,60_ = 25480.39, p < 0.001). As shown, vB_SCh-RP5i3A is the most pH-sensitive phage. It was completely inactivated at pH 2.5 and 3.0 within 1 h. Furthermore, it reduced significantly after exposure at pH 3.5–4.0 for 1–2 h (p < 0.001) (Figure-2a). Likewise, phages vB_SCh-RP60i3A and vB_SCh-RP60i3C were completely inactivated at pH 2.5 within 1 h (Figures-2c and e). However, they were more stable than vB_SCh-RP5i3A as they remained viable at pH 3.0 for 1 h, although completely inactivated after 2 h. The other six phages, vB_SCh-RP5i3B, vB_SCh-RP60i3B, vB_SCh-RP60i4A, vB_SCh-RP60i4B, vB_SCh-RP61i3, and vB_SCh-RP61i4, remained viable at pH 2.5–4.0 for 2 h (Figures-2b, d, f, g, h, and i, respectively).

### Phage morphology

**Figure-3:**
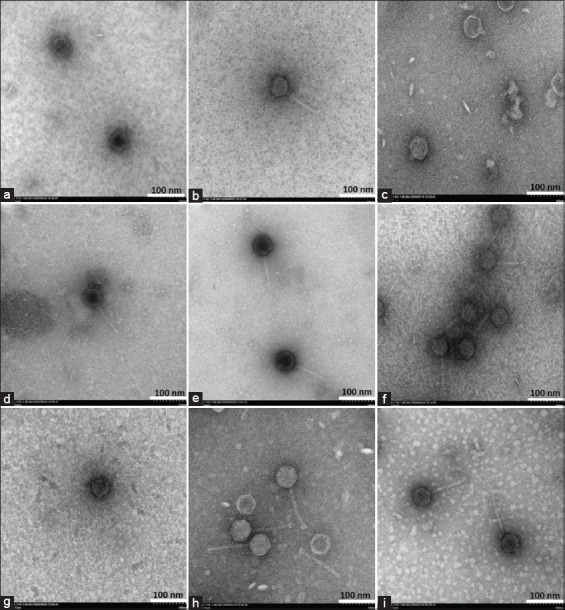
Transmission electron micrograph of phages vB_SCh-RP5i3A (a), vB_SCh-RP5i3B (b), vB_SCh-RP60i3A (c), vB_SCh-RP60i3B (d), vB_SCh-RP60i3C (e), vB_SCh-RP60i4A (f), vB_SCh-RP60i4B (g), vB_SCh-RP61i3 (h), and vB_SCh-RP61i4.

All phages have an icosahedral head (Figure-3). Phages vB_SCh-RP5i3A, vB_SCh-RP60i3A, and vB_SCh-RP60i4B had a short non-contractile tail, as shown in Figure-3a, c, and g, respectively, whereas phages vB_SCh-RP5i3B, vB_SCh-RP60i3B, vB_SCh-RP60i3C, vB_SCh-RP60i4A, vB_SCh-RP61i3, and vB_SCh-RP61i4 had a long non-contractile tail, as shown in Figures-3b, d, e, f, h, and i, respectively.

### Restriction analysis of phage DNA

**Figure-4:**
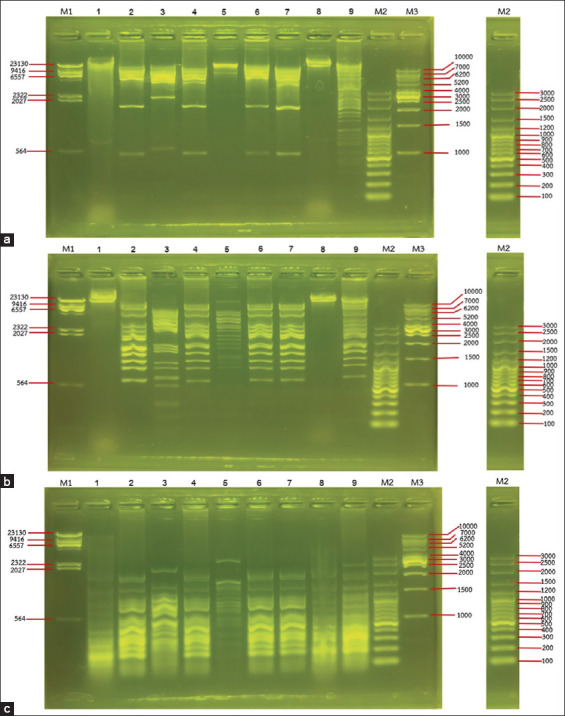
Restriction pattern of the phage DNA digested with restriction enzymes (a) *Eco*RI, (b) *Eco*RV, and (c) *Hin*fI. Lane (1) vB_SCh-RP60i3A, (2) vB_SCh-RP60i3B, (3) vB_SCh-RP60i3C, (4) vB_SCh-RP60i4A, (5) vB_SCh-RP60i4B, (6) vB_SCh-RP61i3, (7) vB_SCh-RP61i4, (8) vB_SCh-RP5i3A, (9) vB_SCh-RP5i3B, (M1) lambda DNA/*Hind*III marker, (M2) VC 100bp Plus, and (M3) VC 1kb Marker.

The genomic DNA of phages was digested by three restriction endonucleases (Figure-4). Based on the results of the restriction digestion, these phages were divided into five groups. Three phages, including vB_SCh-RP60i3C, vB_SCh-RP60i4B, and vB_SCh-RP5i3B, had distinct DNA fragment patterns. vB_SCh-RP60i3A and vB_SCh-RP5i3A displayed similar patterns. The other three phages, including vB_SCh-RP60i3B, vB_SCh-RP60i4A, vB_SCh-RP61i3, and vB_SCh-RP61i4, had similar DNA fragment pattern. Moreover, these results also confirmed that these phages are double-stranded DNA (ds-DNA) viruses.

### The efficiency of phage treatment against *S*. Choleraesuis in SIF

**Figure-5:**
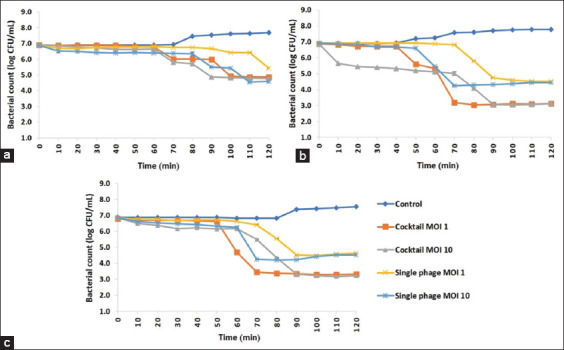
Reduction of *S*. Choleraesuis strains through single phage and phage cocktail treatments in simulated intestinal fluid at the multiplicity of infection of 1 and 10. (a) Reduction of *S*. Choleraesuis KPS585 using vB_SCh-RP5i3B and phage cocktail, (b) reduction of *S*. Choleraesuis KPS604-1 using vB_SCh-RP60i4A and phage cocktail, and (c) reduction of *S*. Choleraesuis KPS615 using vB_SCh-RP61i4 and phage cocktail. Data are means of three replicates with a bar indicating the standard deviation of each time point. *S*. Choleraesuis=*Salmonella* Choleraesuis.

The phage treatment against *S*. Choleraesuis in SIF demonstrated that the individual phages and phage cocktails could reduce the number of *S*. Choleraesuis strains (Figure-5). The number of *S*. Choleraesuis KPS585 with phage vB_SCh-RP5i3B at MOI 1 decreased after 90 min of incubation, whereas MOI 10 decreased after 80 min. However, the number of *S*. Choleraesuis KPS585 decreased after 60 min when treated with the phage cocktail at both MOIs. Furthermore, the number of *S*. Choleraesuis KPS585 with the phage cocktail at MOI 1 showed the highest reduction (2.70 ± 0.02 log CFU/mL) at 100 min compared with its control (*F*_4,10_ = 7023.775, p < 0.001). When the phage cocktail at MOI 10 was used, the highest reduction (2.65 ± 0.02 log CFU/mL) was observed at 90 min (*F*_4,10_ = 4470.234, p < 0.001) (Figure-5a).

In the case of *S*. Choleraesuis KPS604-1 treatment with phage vB_SCh-RP60i4A at MOI 1, the number of bacteria decreased after 70 min. Treatment at MOI 10 lowered the number of bacteria before treatment at MOI 1, which was observed after 50 min. Treating with phage vB_SCh-RP60i4A at MOI 1 showed the highest reduction (3.26 ± 0.03 log CFU/mL) at 110 min compared with its control (*F*_4,10_ = 9089.418, p < 0.001). The highest reduction (3.32 ± 0.04 log CFU/mL) was observed at 70 min when using MOI 10 (*F*_4,10_ = 3605.821, p < 0.001). Interestingly, after treating with the phage cocktail at MOI 10, the number of *S*. Choleraesuis KPS604-1 decreased after 10 min, while MOI 1 decreased after 40 min (Figure-5b).

For *S*. Choleraesuis KPS615 treatment with phage vB_SCh-RP61i4, at MOI 1, the number of bacteria decreased after 70 min, while at MOI 10, decreased after 60 min. The highest reduction of *S*. Choleraesuis KPS615 with the individual phage at MOI 1 was observed at 90 min (2.87 ± 0.03 log CFU/mL) compared with its control (*F*_4,10_ = 6259.109, p < 0.001). When treating with the phage at MOI 10, the highest reduction (2.57 ± 0.02 log CFU/mL) occurred at 70 min (*F*_4,10_ = 9605.463, p < 0.001). However, when treating with the phage cocktail at MOI 1, the bacterial reduction started after 50 min, and the highest reduction (3.37 ± 0.02 log CFU/mL) was observed at 70 min (*F*_4,10_ = 9605.463, p < 0.001). Meanwhile, the number of *S*. Choleraesuis KPS615 decreased after 60 min when treating at MOI 10, and the highest reduction (4.04 ± 0.03 log CFU/mL) was observed at 90 min (*F*_4,10_ = 6259.109, p < 0.001) (Figure-5c).

## Discussion

Bacteriophages have become increasingly popular as antimicrobial agents because of their natural abundance and ability to target specific bacteria. Furthermore, some phages can kill antibiotic-resistant bacteria [39]. Here, we isolated and characterized the bacteriophages specific to *S*. Choleraesuis associated with swine infection. The efficacy of individual phage and a phage cocktail against *S*. Choleraesuis strains in SIF was also evaluated.

Antimicrobial resistance is caused by the abuse of antimicrobial agents. It can cause harm to both humans and animals. Multidrug resistance has emerged in *S*. Choleraesuis, posing a significant therapeutic challenge in swine [40]. Lynne *et al*. [41] discovered that *S*. Choleraesuis exhibited strong resistance to at least 1 antibiotic (87%) and at least 4 antimicrobials (37.5%). In our study, *S*. Choleraesuis strains, isolated from pig intestines, exhibited resistance to antibiotics, including GEN, ENR, NEO, and KAN. Chang *et al*. [42] also reported that *S*. Choleraesuis, isolated from pigs, is ENR and GEN resistant. In addition, Molino *et al*. [43] revealed that *S*. Choleraesuis strain was resistant to two or more antibiotics. Onyango *et al*. [44] found that *S*. Choleraesuis isolated from swine feces were sulfamethoxazole resistant. Different findings on antimicrobial susceptibility may be attributed to the genetic variability in these strains in different countries. Furthermore, different drug usage during animal production may have different drug resistance effects [41]. It was found that all *Salmonella* strains in our study were susceptible to colistin sulfate. Likewise, Cameron-Veas *et al*. [45] discovered *S*. *enterica* isolates from feces with no resistance to colistin sulfate. Poolperm *et al*. [46] revealed that short-term colistin treatment has been linked to the establishment of colistin-resistant *Enterobacteriaceae* in swine. Colistin-resistant *Enterobacteriaceae* occurred quickly after colistin treatment and quickly faded or was eliminated after termination.

In previous studies, lytic phages against *Salmonella* have been isolated from different sources, including sewage water, environmental sources, feces samples, and farm environmental samples [47–51]. In this study, nine phages specific to *S*. Choleraesuis were isolated from natural water and drainage from slaughterhouses. Yajima and Koottatep [52] observed that fecal sludge and market waste were likely the major sources of *Escherichia coli* and *Salmonella* spp. contamination in the canal water in Thailand. The contamination was also discovered in slaughterhouse wastewater [10]. Therefore, *Salmonella* phages have been found in these environments. The isolated phages showed differences in plaque size and clear plaque surrounded with halos. The growing halos around plaques were produced by phages, indicating the manufacture of depolymerases, enzymes that degrade bacterial exopolysaccharides [53].

Instead of a spot test, the EOP assay was used to assess the phage host range in this study. It was suggested that the spot test is an inappropriate method for selecting phages with a broad host range since the lysis result might originate from abortive infection or lysis from without, which could cause a misinterpretation of the outcome. The EOP assay is essential for defining the efficacy of phage lysis [54]. The EOP results revealed that all phages could infect other *Salmonella* strains except for their host. Some phages could also infect different *Salmonella* serotypes. Conversely, the host resistance system or ineffective phage adsorption into host cells could result in a low EOP of a particular phage [55]. Filippov *et al*. [56] demonstrated that altering the surface molecules of bacteriophage receptors could significantly affect EOP and phage adsorption. Hence, the differences in EOP are likely related to strain-specific receptors. Phages with a broad host range and high infection efficiency are preferable for developing phage cocktails for phage applications.

Phages should be stable in various environments to be used as antimicrobial agents. Temperature is a critical factor that reduces phage infectivity. In this study, all phages were stable at a temperature range of 4°C–45°C. Nevertheless, they reduced significantly at 65°C. Bauer and Evilevitch [57] suggested that phages are inactive at higher temperatures (65°C–75°C) due to the failure of phages to retain the packaged genome. Similar to the previous studies, *Salmonella* phages were highly stable at temperatures below 60°C and more sensitive to higher temperatures [58, 59]. Furthermore, pH is an important factor influencing phage stability. Phages are usually stable in the pH range of 5–9 [60]. In pigs, the gastric pH ranges from 1.15 to 4.0 [61]. However, gastric pH values in suckling piglets or weaning pigs are ≥2.5 [62]. Phage sensitivity to acid conditions is common and may significantly decrease phage titers within the stomach [60]. Exposure to low pH conditions can cause irreversible damage to phages. This could reduce the efficacy of phage treatment in the animal’s gastrointestinal system [63]. Yin *et al*. [64] revealed that the phage, PNJ1901, was inactivated at pH 2 and 2.4 after 15 and 30 min of incubation in SGF, respectively. Furthermore, Ramirez *et al*. [65] demonstrated that phages were reduced to undetectable levels at pH 2.4 after 30 min. In this study, most phages remained viable at low pH (pH 2.5–4.0) for 2 h in SGF. These phages appear to be promising candidates for use in treating animals through the gastrointestinal tract.

The morphological characterization of phages revealed that all isolated phages have an icosahedral head and tail. Ackermann [66] reported that more than 96% of identified phages have tail and ds-DNA, with capsids ranging from 30 nm to 160 nm and tail ranging from 10 nm to 800 nm. Three phages in this study had a short non-contractile tail, while six had a long non-contractile tail with an icosahedral head. These morphological variations suggest distinct host recognition mechanisms in host infection [67]. The restriction fragment length polymorphism (RFLP) can be used in the elementary grouping of phages with ds-DNA genomes. The phages with the same restriction pattern from at least three enzymatic cuttings may be classified as the same phages or have a close relationship. However, other characteristics need to be considered. According to our results, the phages that were classified into the same group by RFLP had distinct morphological and EOP results, indicating that they differed. Nevertheless, genomic analysis is necessary to identify these phages.

In this study, the reduction of bacterial cells through phage treatment in SIF was determined to assess the efficacy of phages before their *in vivo* use. In this study, single phages and phage cocktails were used to reduce *S*. Choleraesuis in SIF. However, the phage cocktails were more effective at reducing all *S*. Choleraesuis strains than individual phages. Several studies have shown consistent findings [68–71]. Phage cocktails may remedy the problem of a narrow host range. Furthermore, it could slow down the development of phage-insensitive mutants since different phages can infect the same species and strains [72, 73]. In addition, phage cocktails of more than 2 phages with different cell receptors may aid in slowing bacterial resistance to phages [74]. Furthermore, Bai *et al*. [75] reported that cocktails of three phages inactivated host growth in more than 2 phage cocktails and individual phages. This study’s results revealed that neither a single phage nor a phage cocktail causes phage-resistant bacteria under the conditions of the experiment. As previously reported, no difference exists in the effectiveness between phage cocktails and single phages. However, phage cocktails yielded lower resistance development rates than single phages [76]. Thus, phage cocktails appear to be the most promising option for use as a biological control agent against *Salmonella* in animals.

## Conclusion

This study isolated *Salmonella*-specific phages from natural water and drained liquid samples. These phages could lyse all three strains of *S*. Choleraesuis and *S*. Rissen. Furthermore, they could survive at various temperatures and at low pH. The phage cocktail of the three phages (vB_SCh-RP5i3B, vB_SCh-RP60i4A, and vB_SCh-RP61i4) reduced *S*. Choleraesuis more effectively than individual phages in the artificial intestinal fluid condition. These findings suggest that this phage cocktail is a promising biocontrol agent against *S*. Choleraesuis in pigs through oral administration. However, further *in vivo* studies should be performed.

## Authors’ Contributions

PS and RN: Designed the study. PS: Performed all the experimental procedures. NI: Provided technical help during the experiments. PS: Drafted the manuscript. PS and RN: Revised the manuscript. All authors have read and approved the final manuscript.
